# Use of community resources as health assets for rehabilitation of people with Long COVID in northeastern Spain two years after the outbreak of the COVID-19 pandemic: qualitative study

**DOI:** 10.1186/s13690-023-01139-7

**Published:** 2023-07-06

**Authors:** Mario Samper-Pardo, Natalia Formento-Marín, Bárbara Oliván-Blázquez, Sandra León-Herrera, Belén Benedé-Azagra

**Affiliations:** 1 Institute for Health Research Aragón (IIS Aragón), C. de San Juan Bosco, 13, Zaragoza, 50009 Spain; 2 Department of Psychology and Sociology, University of Zaragoza, Calle de Violante de Hungría, 23, Zaragoza, 50009 Spain; 3 Research Network on Chronicity, Primary Care and Health Promotion (RD21/0016/0005), Carlos III Health Institute, Avda. de Monforte de Lemos, 5, Madrid, 28029 Spain

**Keywords:** Community resources, Health assets, Rehabilitation, Long COVID, Qualitative study

## Abstract

**Introduction:**

The epidemiology of Post COVID Condition is not yet known. There are different treatment options, but they are not recommended or suitable for all those affected. For this reason and due to the lack of health treatment, many of these patients have tried to carry out their own rehabilitation through the use of community resources.

**Objective:**

The objective of this study is to deepen into the understanding about the use of community resources as assets for health and rehabilitation by people with Long COVID and their utility.

**Methodology:**

A qualitative design was carried out with the participation of 35 Long COVID patients, of which 17 subjects were interviewed individually and 18 of them were part of two focus groups. The participating patients were recruited in November and December 2021 from the Primary Health Care centers and through the Association of Long COVID patients of Aragon. The research topics were the use of community resources, before and after their infection by COVID-19, rehabilitation through their use, as well as barriers and strengths for their employment. All analyses were performed iteratively using NVivo software.

**Results:**

Long COVID patients who have used community resources for rehabilitation have seen an improvement in their physical and mental health. Most of them, specifically those affected, have used green spaces, public facilities, physical or cultural activities and associations. The main barriers identified have been the symptoms themselves and the fear of reinfection, with the main advantage of these activities being the perceived health benefits.

**Conclusion:**

The use of community resources seems to be beneficial in the recovery process of Long COVID patients, so it is necessary to continue delving into this topic and promote the formal use of the Recommendation of Health Assets from Primary healthcare.

**Table:** 

Text box 1. Contributions to the literature
• The formal social prescribing by Primary Health Care in Spain has been little studied, so these results are useful for professionals to implement it.
• As far as we know, there are no studies that have delved into the use of community resources as a rehabilitation method for Long COVID patients.
• The findings of this study contribute to the validation of social prescribing as a rehabilitation tool, specifically for Long COVID patients.

## Introduction

In October 2021, the World Health Organization (WHO) established the official definition of “Post COVID-19 condition” [1], more commonly known as “Long COVID”. It is regarded as a novel syndrome with multisystem involvement, characterized by a varied range of both physical as well as neuropsychiatric symptoms. These can be persistent or cyclical, lasting weeks after being infected with COVID-19 [2–4]. The epidemiology and pathophysiology of the disease, as well as the resulting complications, are not known in great depth [5]. As a result, the multidisciplinary approach required for the comprehensive care of patients will likely become one of the biggest challenges for health and welfare services in the coming years [6]. In this regard, the Long COVID forum group has declared that its present and future lines of investigation into Long COVID will focus on its clinical characteristics, through researching and developing treatments [7].

At present, the treatment options available for patients are limited. Only rehabilitative treatments have been shown to be effective in improving the symptoms of Long COVID since no large-scale experimental studies have been conducted on the effectiveness of pharmaceutical drugs to alleviate symptoms [8]. With regard to the rehabilitation offered to patients, various studies suggest that early rehabilitation is vital for overall improvement and better long-term functionality [9, 10]. By contrast, state that the starting point of rehabilitation should be established with caution since in some cases it may cause irreversible harm, thus meaning it is not appropriate for all patients [11]. The types of rehabilitation for Long COVID patients are judged to be similar to those for chronic fatigue syndrome, i.e., those physical and respiratory in nature. Although cognitive behavioral and graded exercise therapies have also been considered necessary, these have caused relapses in some patients [12–15]. Furthermore, this group of patients has required professional care for their mental health, due to the negative effect the disease itself has had on the body and vital aspects of their lives [16, 17].

The need to rehabilitate and treat Long COVID patients leads us to consider the current state of the Spanish National Health Service (SNS) in the wake of the pandemic since the beginning of 2020. Specifically, during the first months of the pandemic, the SNS collapsed and was immersed in a major crisis characterized by a lack of resources in both material and personnel. This made it necessary to develop ethical guidelines for action [18–20]. Over two years later, different services provided by SNS such as recovery and rehabilitation and monitoring and waiting times have been adversely affected and have failed to meet demand [21, 22]. In the context of care for Long COVID patients, in addition the above scenario, there is a lack of knowledge regarding the disease and a scarcity of management guidelines for patients. Taking this all into account, we must consider other courses of action.

With the aim of widening the search for alternative rehabilitation treatments for patients, a seldom studied trend first identified in the UK appears to be gaining momentum. The technique, known as “Social Prescribing”, is being used by primary health care (PHC) centers as a method of rehabilitation for Long COVID patients. However, only one qualitative study conducted in the UK has assessed the effectiveness of the tool, which promotes the use of social prescribers, as well as the involvement and strengthening of community support services [23].

In Spain, the social prescribing technique has been rolled out in a number of regions for some years, even though it is still under development. In fact, one of the objectives of the “Action Plan for Primary and Community Healthcare 2022-23”, published by the Ministry of Health in Spain, is for it to be accessible in every region. The tool was established in the country under the adapted name, “Recomendación de Activos para la Salud” (Recommendation of assets for health) or RAS. Its name is present in regional plans around the country (In Aragon, Andalusia, Asturias etc.) RAS calls for the creation of different formal mechanisms to prescribe non-clinical alternatives to patients under the PHC umbrella, which have a positive impact on their health. It is a multidisciplinary technique, with health as the underlying focus, which allows individuals and companies to have the necessary means to improve their health [24–27] .

In this regard, a health asset can be defined as being “any factor or resource that enhances the ability of individuals, communities and people to look after their health and wellbeing.” They have the ability to improve the circumstances of individuals or groups, improve or look after their physical, mental and social health and deal with stressful situations [28]. For this reason, health assets are general resources used to deal with difficulties and inequalities, as well as enhance capacities and skills towards what enables individual and collective health and empowerment to overcome difficulties in the face of inequality. It is essential to focus on skills and abilities that enhance health, improve self-esteem and individual and collective empowerment [29, 30].

For all of these reasons, given the challenge of providing a response to Long COVID patients and the lack of evidence on RAS as a rehabilitation strategy for these patients, this article aims to generate scientific evidence in this field, in order to avoid the process of excessive medicalization as well as relieving the pressure on the rehabilitation services of the SNS.

Hence, the objective of this study is to deepen our understanding of the use of community resources as assets for health and rehabilitation by people with Long COVID and their usefulness.

## Methods

### Study design

A qualitative study based on interviews in deep and focus groups was carried out, through the thematic analysis based on the grounded theory, of an inductive nature [31]. Qualitative methods are optimal for delving into human experiences such as emotions, attitudes and expectations [32]. For this reason, this methodology was chosen in order to collect subjective information and access the perceptions and experiences of Long COVID patients, in relation to the use of community resources as health assets. The intention of conducting in-depth interviews was to be able to argue from calm. However, focus groups were conducted as interpersonal interactions can generate answers and insights that did not emerge during in-depth interviews [32]. The authors followed the consolidated criteria for reporting qualitative research (COREQ) checklist.

The results obtained in this study contributed to the design of a randomized clinical trial called: “Analysis of symptoms and quality of life of people with a prolonged diagnosis of COVID-19, and the efficacy of an intervention in primary health care using ICT” (ISRCTN91104012), registered on 10/02/2022 [33].

### Sampling and sample size

The inclusion criteria of the participants were the following: being over 18 years of age and having been diagnosed with Long COVID by a general practitioner (GP) or PHC specialist. The exclusion criteria were the following: not being able to respond to the interviewer for any reason, presenting high cognitive impairment for any reason and/or receiving palliative care.

An intentional sampling strategy [34] was carried out among patients diagnosed with Long COVID treated in seven PHC centers in the province of Zaragoza (Northern Spain), and also from the “Long COVID Aragón” Patients Association. A recruitment time of twenty days was established, during the month of November 2021. Recruitment was carried out by the GPs themselves, who volunteered after a meeting with a member of the research team, in which the objectives of the project were explained. Each GP made a list of potential patients, using purposive sampling to obtain a heterogeneous sample and to be able to explore the topics of interest with breadth and depth [35]. Subsequently, each GP made face-to-face contact with each of the possible participants to verify which met the selection criteria. The GPs were provided with information documents about the study, which they could offer to interested patients, in which a telephone number appeared where they could obtain more information and confirm their wish to participate. When a potential patient contacted the research group, a researcher (SL-H) is in charge of resolving possible existing issues and making an appointment in person at the research group’s headquarters, located in a PHC center in Zaragoza. Once the face-to-face meeting took place, the same researcher (SL-H) re-verified that the potential participant met the selection criteria and proceeded to sign the informed consent.

The research team established that the final sample size would depend on information saturation, established as the point at which no new information was extracted. Initially, a total of 39 subjects were interested in participating in the study. Finally, the sample size consisted of 35 participants, since 4 patients refused to participate due to the incompatibility of schedules to attend the interviews. Information saturation occurs when no new categories emerged after analysis of focus group data [35]. In this case, the second focus group did not provide new categories, so it was concluded that information saturation had been achieved. In this way, it was not necessary to start new recruitment processes.

### Participant’s characteristics

A total of 35 subjects participated in this study, 17 of them were interviewed individually and 18 took part in two focus groups, nine in each group.

Regarding their sociodemographic characteristics, 71.4% were women, the mean age of the participants was 49 (SD: 10.81) and the mean number of months elapsed since COVID-19 infection was 14.80 (SD: 3.90). Table 1 shows the main characteristics of the participants in terms of age, sex, marital status, educational level and employment status. This sampling was used to analyze differences between different patient profiles.

**Table 1:** Sociodemographic characteristics of participating patients; Zaragoza, 2021

Variables	Patients (n = 35)
*Age*	
20–40	8 (22.9%)
41–60	20 (57.1%)
> 60	7 (20%)
*Sex*	
Male	10 (28.6%)
Female	25 (71.4%)
*Marital Status*	
Single	4 (11.4%)
Married or in a couple	19 (54.3%)
Separated or divorced	10 (28.6%)
Widowed	2 (5.7%)
*Education level (%)*	
No formal education but can read and write	1 (2.9%)
Primary education	3 (8.6%)
Secondary education	19 (54.3%)
University education	12 (34.3%)
*Employment status (%)*	
Employee	6 (17.1%)
Employee with TWD	22 (62.9%)
Unemployed with benefits	1 (2.9%)
Unemployed without benefits Retired	1 (2.9%)
	5 (14.3%)

TWD: temporary work disability

### Data collection

All interviews and focus groups were conducted by a moderator (MS-P) and an assistant (NF-M); both PhDs, graduates in social work and nursing, with previous experience and specific training in qualitative methodology. The moderator and the assistant introduced themselves to all the participants as project researchers in charge of conducting the interviews and focus groups. None of the members of the research team were related to the participants.

All sessions were held during the months of November and December 2021, both in the morning and in the afternoon, in order to facilitate availability. They were held in a room attached to the PHC center, with an independent entrance, with the aim of creating an environment for discussion, away from the clinical context of the PHC services. It was a neutral room so that the participants did not feel conditioned or uncomfortable. During the interventions there was no person not interviewed, in addition to the two researchers mentioned.

A standardized protocol was planned to guide individual and group interviews. A topic list to be addressed during the interviews and focus groups was prepared, as shown in Table 2. The topic list was based on the previous bibliography [36–39] and the clinical experience of the research team. The objectives of the study were addressed indirectly and the questions asked about the topics were answered openly and progressively. The interviewer and the moderator assumed a minimal role in the orientation and limited their interventions to address the themes of the script. First, individual interviews with patients were conducted, followed by two group interviews until information saturation was obtained. No prior pilot interviews were conducted. In-depth interviews lasted between 20 and 60 min and group discussions lasted between 50 and 75 min. It was not necessary to repeat any interview, nor was there any interruption during the recordings. All sessions were digitally audio-recorded and transcripts of these records were obtained to compose a final set of qualitative data for analysis.

**Table 2:** Topic list and questions for patients. Zaragoza, 2021

Topic list	Questions for patients
Before the interview	1. Welcome, acknowledgements, and introduction of the interviewer and observer. 2. General information about the topic to be discussed and the purpose of the session. 3. Explanation of the dynamics of the interviews regarding ethical issues (confidentiality and informed consent and permission to record), and the functioning (the interest is about the participants´ opinions, there are no right or wrong answers).
Assets for health in people with Long COVID	Rehabilitation activities or resources you use that help you improve your state of health. Was it on your own initiative?
Patients´ awareness of community resources	Knowledge of community resources is health assets and rehabilitation assets. Benefits (if any) of using community resources Availability of community resources
Barriers and strengths regarding the use of community resources	Barriers Strengths
Adequacy of community resources to meet the needs of people with Long COVID	Are there community resources that satisfy the needs of people with Long COVID?

### Data analysis

The transcription of the interviews and focus groups were carried out verbatim by two external researchers, with previous experience in carrying out this action. The names of the participants were anonymized with an assigned numerical code. Some participants reviewed the transcripts, approving them and, finally, the field notes made during the interviews were added.

Thus, the grounded theory approach was employed for data analysis [31]. Data collection, analysis, and axial theoretical coding were performed using a constant comparison process [40].

All analyses were performed iteratively using Nvivo software. Two authors (BO-B and BB-A) reviewed the transcripts independently, coding the sentences that contained significant units of analysis. These were grouped into categories, through a combination of emerging codes. The same two researchers reviewed and compared their findings, reaching an agreement on codes and categories. Two rounds of coding and discussion were carried out to achieve clearer categories and improve the reliability of the process. This process was iterative with subsequent transcripts. No new categories emerged at the end of the second focus group, implying that information saturation had been achieved. Subsequently, a grouping of categories was carried out and these, in turn, into subcategories based on the uniformity of themes and subthemes of a higher conceptual level.

To check for consistency, the moderator (MS-P) and the assistant (NF-M) in all interventions checked their agreements by blind review [40]. Any disagreements between the two investigators were resolved by discussion.

At each step, an independent author (RM-B), acting as a reviewer, verified that the data consistently supported the analyses, in order to improve reliability and transferability [34].

Finally, axial coding was performed. The categories that emerged in the previous step were reorganized creating new relationships between the concepts. Among all the categories that emerged in the first phase of open coding, those that seem most interesting are selected to delve into their explanation [41]. This action was carried out by three researchers (MS-P, BO-B and BB-A) until a final agreement was reached.

## Results

**Fig. 1:**
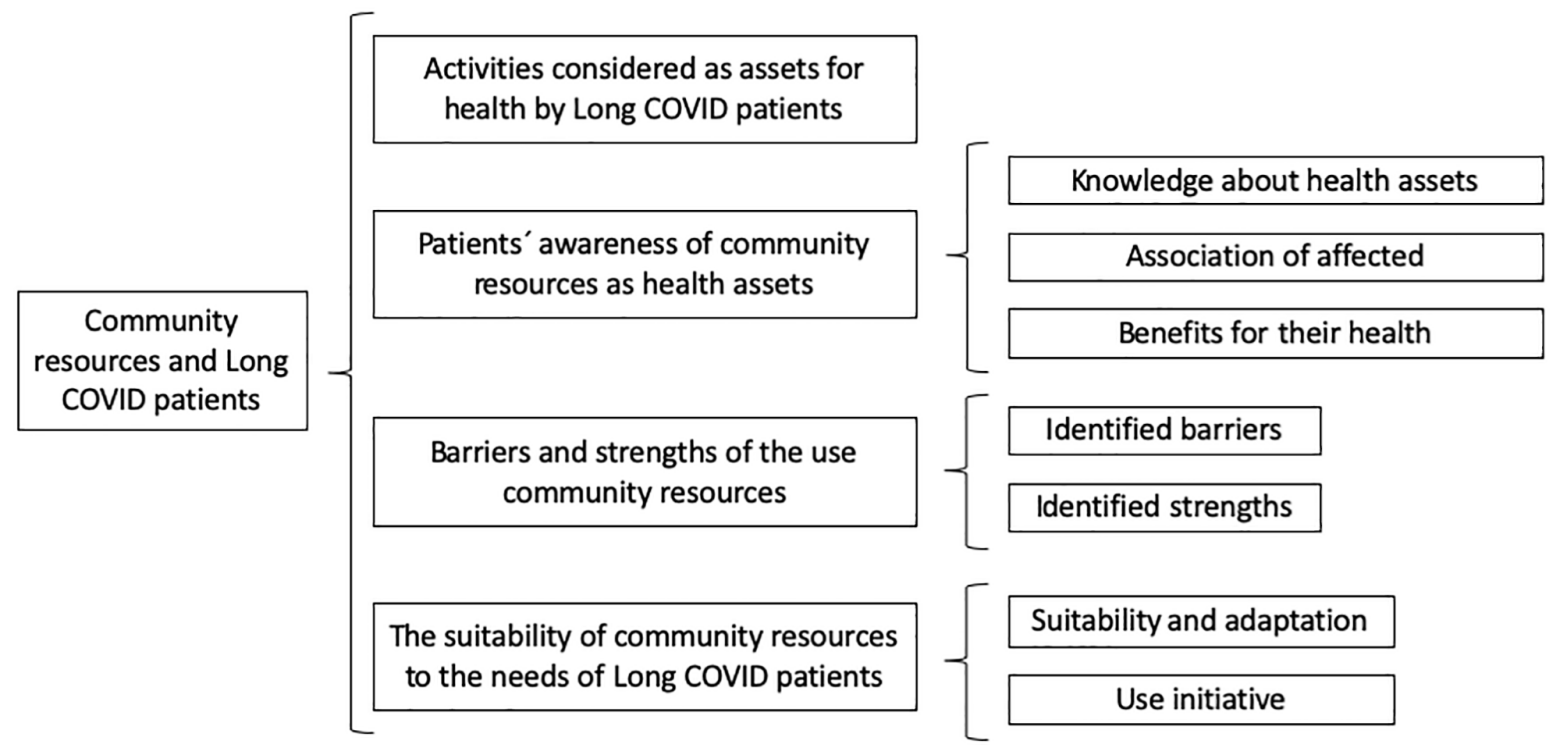
Graphic representation of the central aspects of the results; Zaragoza, 2022

As shown in Fig. 1, a total of four main themes were obtained: (1) Activities considered as assets for health by Long COVID patients; (2) Patients’ awareness of community resources as heath assets; (3) Barriers and strengths of the use of community resources; and (4) The suitability of community resources to the needs of Long COVID patients. In addition, a total of seven subcategories were identified.

### Activities considered as assets for health by long COVID patients

The majority of the patients undertook activities that are considered beneficial for their health if their physical condition allowed them to. These activities, which count as health assets, mainly comprise physical exercise, walking and also cognitive stimulation activities via Internet apps. Many of these activities done during rehabilitation make use of community resources, such as green spaces and swimming pools. A smaller number of patients consider participating in group community activities as a health asset, but the main activities identified were memory workshops, Nordic walking groups, Pilates and yoga. It should be noted that most of these activities were carried out on the patient’s own initiative, without the involvement of the health system. This is especially true of PHC, which is the closest to the community with regard to the social prescribing of community resources.

*I walk a lot now in the park or I go out cycling.*

(Male, 56)

*I am enrolled in an organization that offers memory workshops. The problem is that they are designed for older people. (Male, 40)*

*Here in my neighborhood for example, they do yoga in the civic center and I did look into it but I thought I wouldn’t be able to keep up. (Female, 44)*

*I have been going to yoga sessions at the cultural center in my town for two months. It is going very well because I was not very good at the breathing techniques, and the truth is that it is noticeable. Also the stretching, the positions, etc.*

(Male, 60)

### Patients´ awareness of community resources as health assets

The majority of the participants identified parks, public transport, sports facilities and cultural activities organized by city councils etc. to be community resources. There are people who have a better knowledge of community resources, and who are overall more involved in the community and community activities. Furthermore, those that participate the most in community activities are retired or semi-retired people, who began to use community resources after finishing their careers.

When talking about community resources which are health assets, the majority of the participants said that they use parks and green spaces for Nordic walking or regular walking. People who had been involved the most in the community before their bout with Covid-19, say that they participate in other activities offered by local governments or neighborhood associations. This group considered these activities to be health assets to improve their symptoms. An example of these activities includes theatre, as they are working on their memory by memorizing scripts, an aspect which affects a large percentage of Long COVID patients. Other types of activities which stand out are yoga and Pilates. There are also patients who have enrolled on these types of community activities to improve their health, who did not do so previously. They report that they have found them to be very beneficial not only for the physical benefits but also in a psychological sense.

*Some resources yes, maybe the bus sometimes, the parks for walks, sports facilities no. But I have been to the library sometimes. I’ve never been to local government activities.*

(Male, 56)

*I sometimes go to cultural centers, public parks because I walk (…) I am in two organizations in which I am very active. I belong to the 8 M assembly (Feminist society) where we have meetings and activities (…) I am also a member of COESPE (Pro-public pension pressure group) and we are very active… I am also retired, I live alone and do not work… it is not that I feel alone, but it is interesting to participate and do things with other people. It is productive and beneficial for both my physical and mental health.*

(Female, 71)

*I sought out yoga. I was a bit nervous as I hadn’t done these sorts of activities before and the truth is it is going very well. I am more aware of my breathing and it helps me to relax.*

(Male, 60)

There are patients who are members of their regional Long COVID Association and see it as a very valuable community resource and health asset. The association stepped up to respond to the needs of its members. As they say, there are people who have been involved since it was founded and it has helped them be active, and participate in activities. It also provides mental and social support and gives them hope in fighting the disease.

*Yes, I am part of the Long COVID group in Aragon, this has also helped me a lot. Apart from the fact that they understand you, you don’t have to explain things, we support each other. It is making me very active because I am quite involved. I am following all the topics, and reading everything that the scientists are sending me, news press releases, I am taking advantage of it a lot.*

(Female, 44)

*For me, there were some turning points. One of those was becoming aware of the ‘Covid Persistence Collective’*.

(Female, 64)

There is a general perception that the community resources they are using are helping them in their rehabilitation and state of mind.

Regarding the availability of community resources, in the urban environment, there is a perceived large availability of community resources. However, in the discourse, there is a lack of knowledge regarding them. Patients who live in rural areas, depending on the size of the town, say that they do not have the community resources, such as indoor swimming pools, that could be health assets. Therefore, they depend on having a car or public transport. Additionally, if they do have a car, they are not in a fit state to drive.

*Everything can be improved. If we could have a heated swimming pool, it would be great for me. For example, if I want to go to a swimming pool to do exercises in the water, I would have to drive 30 km. Another thing is that driving is difficult because you lack reflexes.*

(Female, 70)

### Barriers and strengths of the use community resources

As with barriers to the use of community resources as health assets, a declining state of health is mainly apparent in the discourse and fatigue is repeated as a main barrier.

*At first, I was thinking of signing up for Yoga, but I get tired as soon as I do anything.*

(Female, 44)

Another barrier that appears in the discourse is the fear that a new disease could cause a relapse in their health. Some people have improved their health status and they are scared of going back to the start and losing all of their improvement. On the other hand, aside from their physical and mental health, they have isolated themselves from society and are finding it difficult to rejoin. Lastly, it is apparent that feeling like a burden on others is also a barrier.

I notice that I feel I am very far away from ‘normal’ society

(Female, 42)

*We have a fear of being infected again, know how it is… with a relapse, getting infected again I think it would ruin us… apart from being emotionally devastating, it could also be very dangerous physically.*

(Male, 48)

*I have realized that it has to be done step by step at my own pace because if I start doing things with a group of people, I will not be able to follow them. This would affect me mentally.*

(Female, 70)

As a strength, they highlight their closeness and the fact that they are aware that they will be useful for their physical, mental and/or social condition.

*When the local government organizes activities, I try to go in order to get out of the house and socialize a bit.*

(Female, 50)

### The suitability of community resources to the needs of people with long COVID

With regards to suitability, they are community resources that are generally viewed as useful in the recovery process. Notable examples are walking in parks and green spaces and yoga, which helps some people to be aware of their breathing. Other examples include memory workshops offered by local governments and elderly peoples’ associations, however, the perception is that they need to be more adapted to their own specific needs (e.g. there is a marked deficiency in terms of verbal fluency).

However, it is the patients themselves who think about what community activities on offer can be useful to them during their rehabilitation. Only a few patients have had a health asset recommended to them by either PHC or mental health professionals. Normally it is the patient who looks for activities according to their needs or they are advised by friends and family. They try to participate in community activities as an additional treatment during their rehabilitation.

*We are in a neighborhood association and memory workshops but they are not designed for us, they are designed for elderly people with differing needs. (Female, 38)*

Everything could be improved, and for patients with Covid, that goes without question. (Male, 62)

## Discussion

This study is the first source of evidence in Spain on how the use of community resources, as a rehabilitation method, can improve physical and emotional well-being in patients diagnosed with Long COVID.

Scientific literature has demonstrated the benefits of health assets to improve physical and mental health [27, 37]. Some authors conclude that patients with general health problems and regularly in PHC consultations could benefit from social prescribing, thus decreasing the use of the SNS, making it a cost-effective alternative for the management of long-term conditions [42–44].

Among the main results of this study, it is noteworthy that most of the patients used a community resource, many rehabilitative in nature, to improve their physical and/or cognitive capabilities. The main motivation reported by the participants was to seek an improvement of the negative health effects arising from being infected with COVID-19 and its progression. Those who have used community resources reported improved physical ability and emotional management. Similarly, it has been shown that patients perceived that social prescribing increased their self-esteem and self-efficiency because they were able to access the help they needed and develop support networks [42]. This highlights how Long COVID patients were able to manage their health using community resources in their surroundings to meet their new needs which were not covered by the healthcare system. Social prescribing, therefore, appears to be a useful tool in addressing persistent symptoms for these patients.

Another finding of the study, which is already present in the literature [27, 45], is that people with great ties and participation in their community before being infected with COVID-19, have greater knowledge of the resources at their disposal and make greater use of them. Therefore, strengthening these two aspects among the general population would have a positive impact on how people manage their own health. The factors influencing the effectiveness of social prescribing depend on the person’s experiences with their referents, the type of activities on offer, the needs of the patients and the benefits for their health and well-being after the use of the resources [44].

The types of resources identified were mainly green spaces, local facilities, physical and cultural activities and societies. These types of resources are among the main types mentioned in the literature [46]. With respect to the availability of community resources, people living in urban environments report a greater availability of resources than those in rural areas. As established by the Spanish Ministry of Health’s guidelines on community health [47], as well as inequalities in health, geographical inequality also exists. Those living in urban areas are advantaged over rural populations, with the latter being forced to travel to nearby localities.

Most of the health assets were suited to the patient’s needs. Cognitive stimulation activities were an exception, as these cater for elderly patients with differing needs. In order to increase community engagement, it is essential to promote participation and collaboration between different entities in the community [48]. Collaborative work between social and health professionals and patients and community resources will enable the development of resources appropriate to the emerging needs of the population.

For social prescribing to be effective, patients have to be appropriately transferred from PHC to a relevant resource [44]. Regarding how they became aware of resources, the participants indicated that they mostly used their own initiative and, in some cases, they were referred to as a health asset by PHC professionals. In a qualitative study conducted in the United Kingdom, patients with Long COVID demanded greater awareness of available resources. After this need was identified, social prescribing was recommended to these patients through online platforms and by healthcare professionals [23].

On the subject of the knowledge of participants’ barriers to using health assets, a number of factors stand out. These include a detreating health condition, fatigue, difficulties in resuming social contact, fear of reinfection and not being able to perform as they did before. Knowledge of these barriers would allow professionals and resources to approach patients to promote social prescribing or RAS. The use of social prescribing had a positive impact on increasing independence in the use of services, participation in community activities, control over their health and an improvement in the management of their health condition [37].

By contextualizing the results obtained, various factors that occurred during the pandemic should be considered. A number of community activities during the months with greater restrictions on gatherings were reduced compared to the pre-pandemic situation, which may have impacted the availability of resources. On the one hand, the lack of referral to community resources by PHC professionals may have been due to the fact that during the pandemic, this area of the health system was responsible for tracking COVID-19 cases and identifying COVID-19 contacts. In Aragon (Spain) specifically, the Community Care Strategy [49] has been in place since 2018. Despite this, however, its development was sidelined by other activities during the early years of the pandemic. On the other hand, regarding the identification of health assets, it should be noted that the participants had not received prior training on the asset model, making it difficult to identify resources.

The experiences of this group of patients show that much remains to be done, but that there is hope. Obtaining these results opens the doors to new lines of action. Health care must respond to the health problems of the population, among which are chronic diseases. The SNS could implement different prevention and health promotion strategies centered on a community approach. This requires adequate community infrastructures, in such a way that the involvement and collaboration of government agencies is needed. However, it would be a cost-effective strategy that could alleviate SNS waiting lists, especially after the COVID-19 pandemic [50]. In short, the need to care for Long COVID patients could contribute to the promotion of RAS and social prescribing, understanding it as a formal healthcare service for these patients, and those with similar symptoms.

Regarding the limitations of this study, due to the characteristics of community interventions and social prescribing, most of the studies that present evidence are qualitative in nature. It is, therefore, necessary to increase the number of quantitative studies [51]. Additionally, the symptoms which limit the patients do not come from the electronic medical records of the patient, but from personal accounts provided by the patient, meaning that self-perception bias may be present. With regards to the strengths of this study, it allows us to understand the characteristics of the use of health assets by Long COVID patients in order to promote social prescribing to this group, as there are no previous studies on this group of patients.

## Conclusion

In conclusion, most patients that made use of community resources identified as active. This allowed them to improve their ability and emotional wellbeing and it can therefore be considered a useful tool for addressing the main persistent symptom reported by Long COVID patients. Formal social prescribing or RAS of resources by PHC professionals in Aragon (Spain) has been infrequent, so taking this into account will allow for the development of formal training programs at the institutional level to involve health professionals and encourage them to make use of the tool. Furthermore, exploring the motivations and barriers to using these resources will be useful for professionals to address them and favor the use of community resources for patients.

## Data Availability

The datasets used and/or analysed during the current study are available from the corresponding author upon reasonable request.

## Abbreviations

WHO: World Health Organization
SNS: Spanish National Health Service
PHC: Primary Health Care
RAS: Recommendation of Assets for Health
